# Idiopathic erythema annulare centrifugum successfully treated with doxycycline: A viable therapeutic option?

**DOI:** 10.1111/srt.13477

**Published:** 2023-09-26

**Authors:** Cristian Fidanzi, Matteo Bevilacqua, Giorgia Salvia, Marco Romanelli, Valentina Dini, Agata Janowska

**Affiliations:** ^1^ Unit of Dermatology University of Pisa Pisa Italy

Dear editor,

Erythema annulare centrifugum (EAC) is an inflammatory skin disease with unknown etiopathogenesis.^1^ It is thought to be associated with various pathological conditions (fungal, bacterial, viral and parasitic infections, cancer, Crohn's disease, autoimmune endocrinopathies, hypereosinophilic syndrome), drugs, food exposures, but most of the time, EAC is idiopathic.^1^

We report the case of a 74‐year‐old female who presented to our outpatient clinic with annular erythematous plaques with central clearing, slightly raised edge, non‐scaly that merged into polycyclic lesions scattered all over the entire body surface for 10 months (Figure 1A–C). Incomplete arcuate patches and trailing edges were present at the level of the back. Itching was absent (Figure 1C). In the past, the lesions had rapidly disappeared after a course of oral corticosteroids (OCS) with recurrence upon discontinuation, which did not resolve after a second course of OCS. We performed a skin biopsy of the lesion edge that showed hyperkeratosis and diffuse dermal lymphocytic infiltrates at both perivascular and interstitial level rich in plasma cells, eosinophils and histiocytes (Figure 2). Direct immunofluorescence (DIT) on perilesional skin was negative. The autoimmune panel, routine blood tests and tumor markers were normal. Serological tests for CMV, EBV, borrelia burgdorferi, Helicobacter pylori and stool ova and parasite exam were negative as well. A fecal occult blood test (FOBT) was positive in one out of three specimens, so colonoscopy was performed, which showed only a small polyp that was immediately removed. Abdominal ultrasound and chest x‐ray were negative. The patient had reported no changes in eating habits and had not started any new medications before the onset of this skin condition. Based on the data we gathered, we made a diagnosis of idiopathic EAC, Darier variant. Since no underlying cause was found and previous failure to OCS was reported, we decided to start oral doxycycline 100 mg (one tablet per day after the main meal).^2^ At 40‐day follow‐up only small, mildly itchy urticarial papules of the right upper extremities and abdomen remained while others completely disappeared. Therapy was maintained for another 2 months during which the lesions initially increased in number but then gradually disappeared permanently. At 6 months after completion of therapy, the patient was disease‐free without development of any side effects (Figure 1D–F).

**FIGURE 1:**
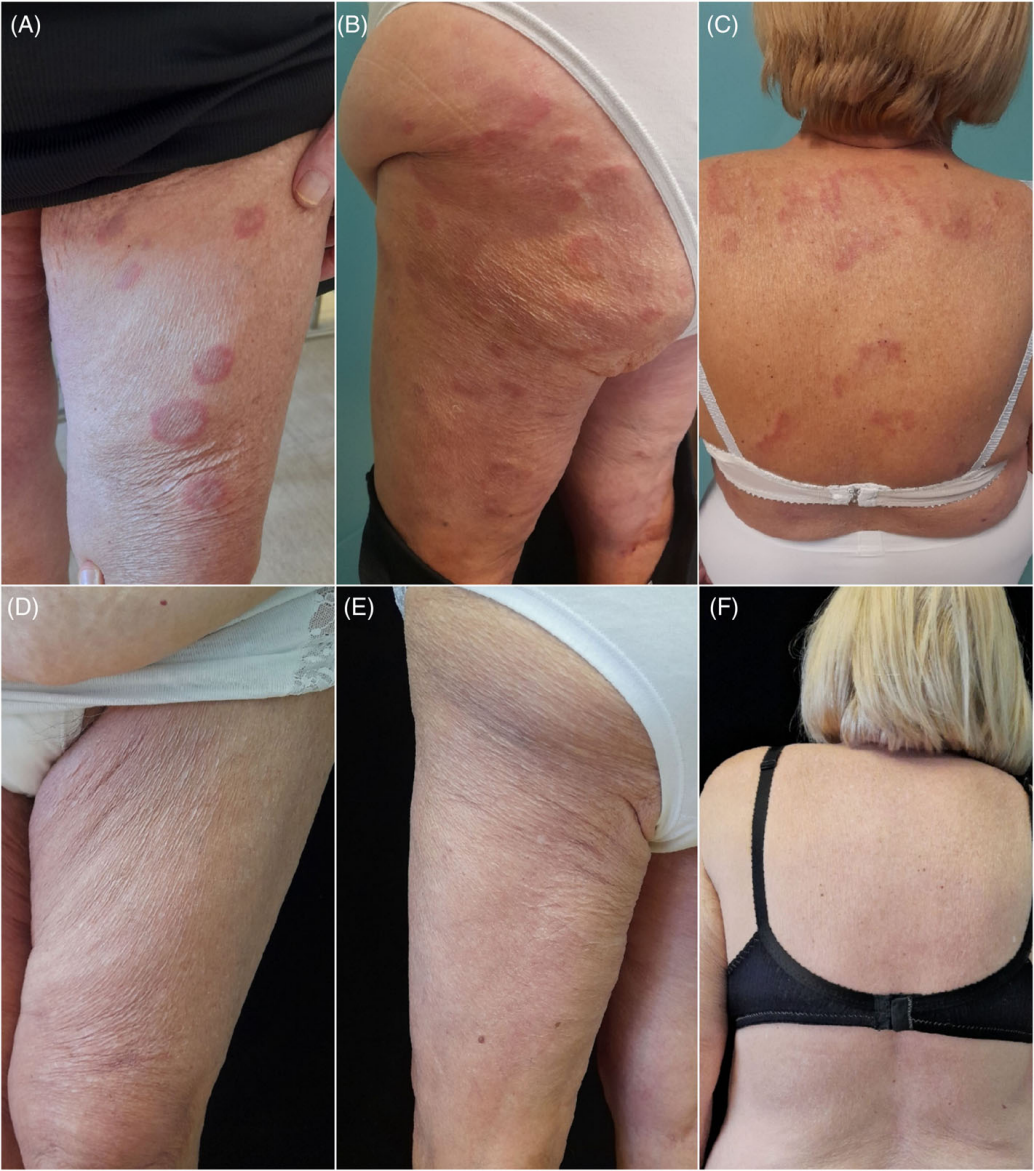
Arcuate erythematous lesions due to erythema annulare centrifugum and subsequent disappearance of the lesions. (A)–(C) Initial clinical image depicting arcuate erythematous plaques with central clearing and advancing edge scattered all over the back and lower extremities. (D)–(F) Complete resolution of the skin lesions after 6 months of therapy with oral doxycycline.

**FIGURE 2:**
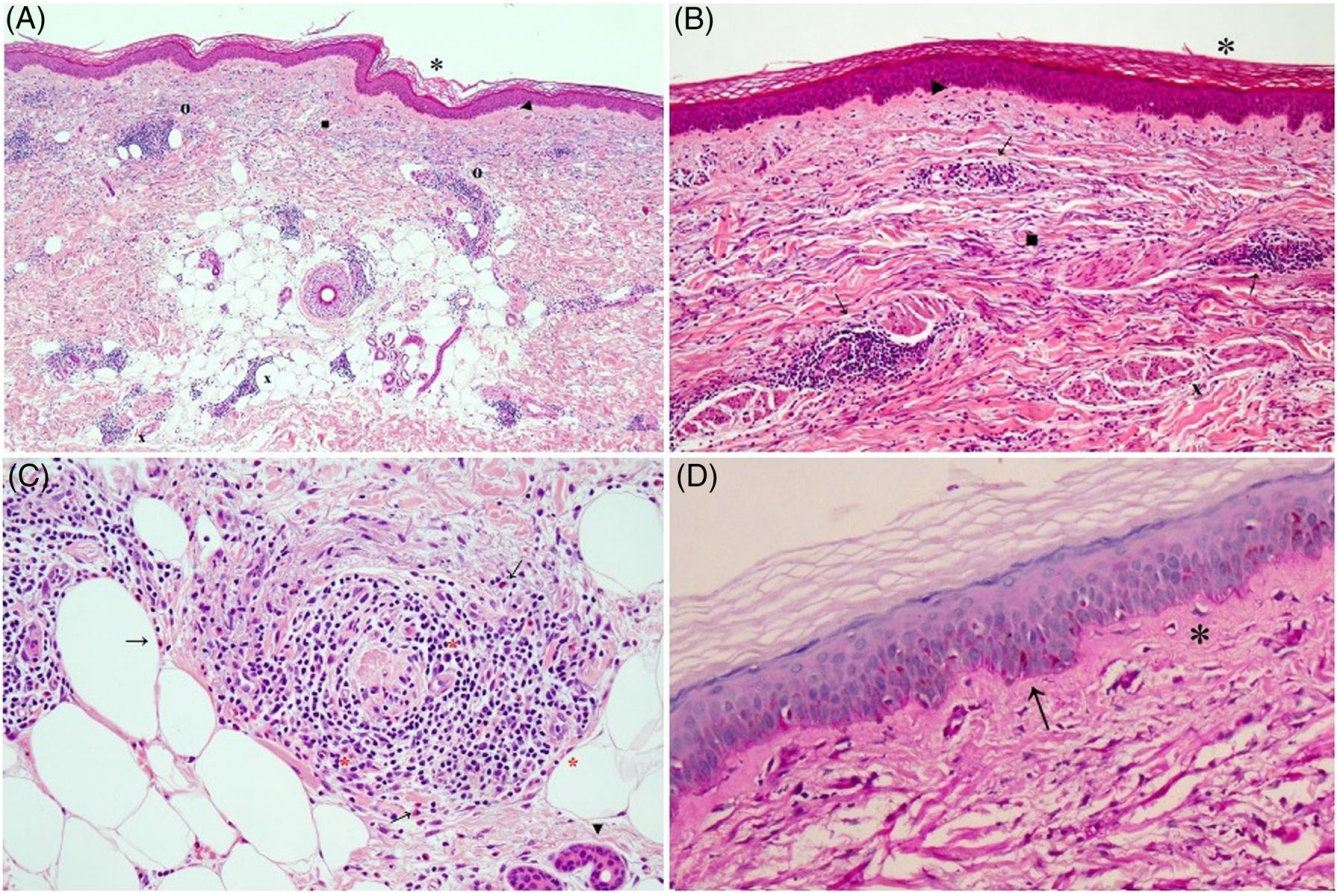
(A) The skin shows diffuse orthokerratotic hyperkeratosis (*) and mild flattening of the dermal‐epidermal junction (►). It can be appreciated actinic elastosis in the dermis (■) associated with scattered perivascular and periadnexal lympohistiocytic infiltrates both in the upper papillary (**o**) and lower reticular dermis at the dermal‐epidermal junction (**x**) (H&E, 5×). (B) Furthermore, a mild periadnexal deposition of mucin is observed e (**x**) periannessiale di mucine (H&E, 10×). **C)** Lymphohistiocytic infiltrate in the lower reticular dermis including plasma cells (*). Eosinophils are present (**→**) (H&E, 20x). (D) Periodic Shiff's Acid (PAS, 20×) staining shows sparse findings of subepidermal fibrosis (*) and epidermal basement membrane (**→**) substantially a normal finding.

Treatment of an associated underlying disease can lead to the resolution of EAC; if a cause cannot be identified, no specific therapies are available.^1^ The use of systemic corticosteroids can induce complete remission of the lesions, although upon discontinuation recurrence is frequent.^1^ Some authors have proposed the empirical use of antibiotics and antifungals because of the possible association with infections.^1^, ^3^, ^4^ In the literature we found at least one case successfully treated with doxycycline,^2^ an antibiotic with anti‐inflammatory activity and usually well tolerated.^5^ It is not possible to know whether the lesions would have resolved spontaneously without therapy or whether this occurred because of the anti‐inflammatory or antibiotic action (on a possible undetected infection) of doxycycline, but certainly this drug represents a therapeutic option that should be considered in the management of idiopathic EAC.

## CONFLICT OF INTEREST STATEMENT

Our research group declares no conflict of interest.

## INFORMED CONSENT

We obtained informed consent from the patient for the use of clinical and photographic data.
